# Supplementary material for: Assessment of Protein Complex Predictions in CASP16: Are We Making Progress?
Source: Proteins. 2025 Oct 31;94(1):106–30. doi: 10.1002/prot.70068 (PMC12750043; doi:10.1002/prot.70068)
Supplement: Supplementary file 1 — Data S1: prot70068‐sup‐0001‐Figures.pdf. [file PROT-94-106-s002.pdf]

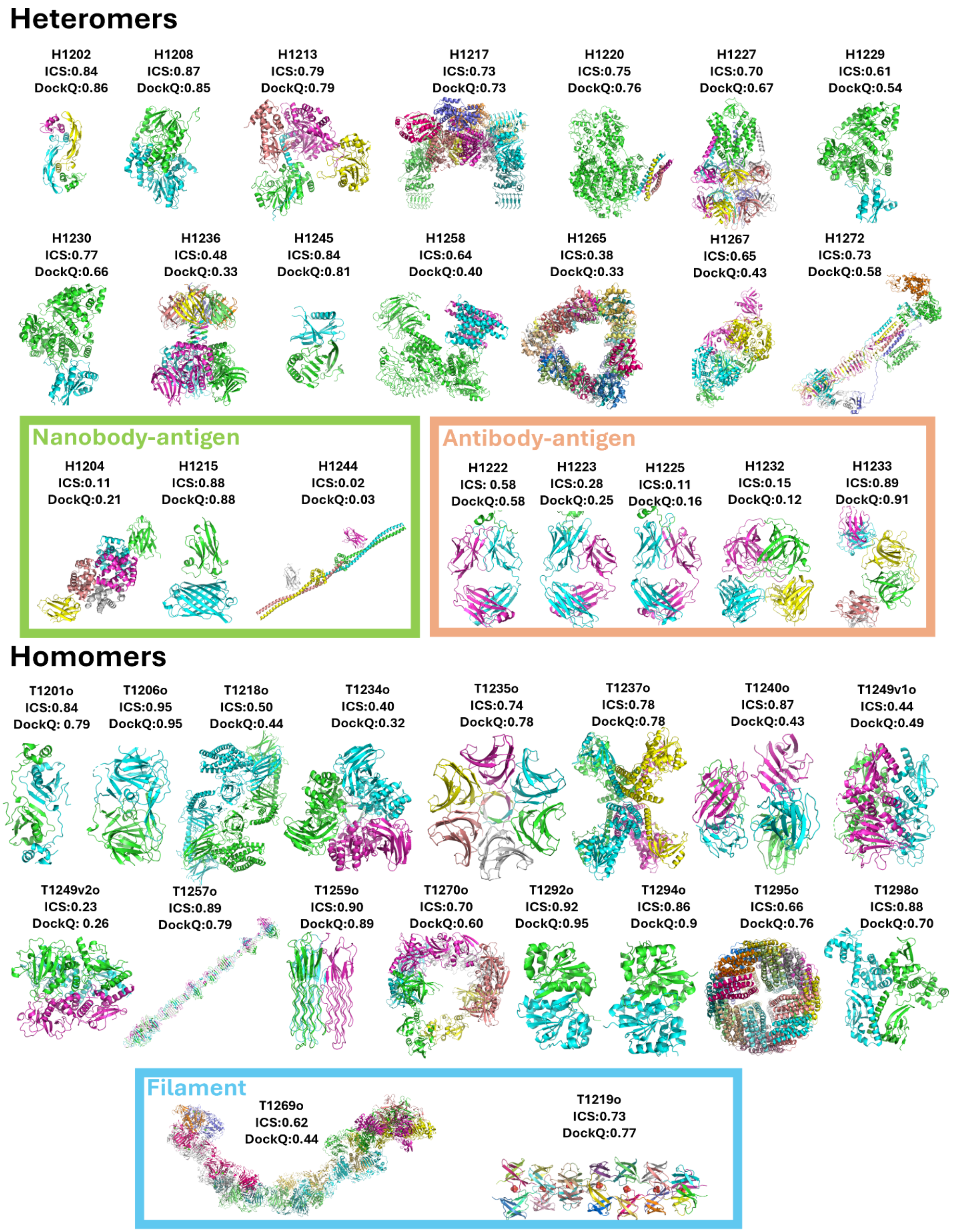

Figure S1. Overview of 40 assembly targets in CASP16, with ICS and DockQ scores in the top 5th percentile for each target. The DockQ and ICS were calculated based on the formula in the "Methods" section and thus may be different from values provided by organisers on CASP16 official website.

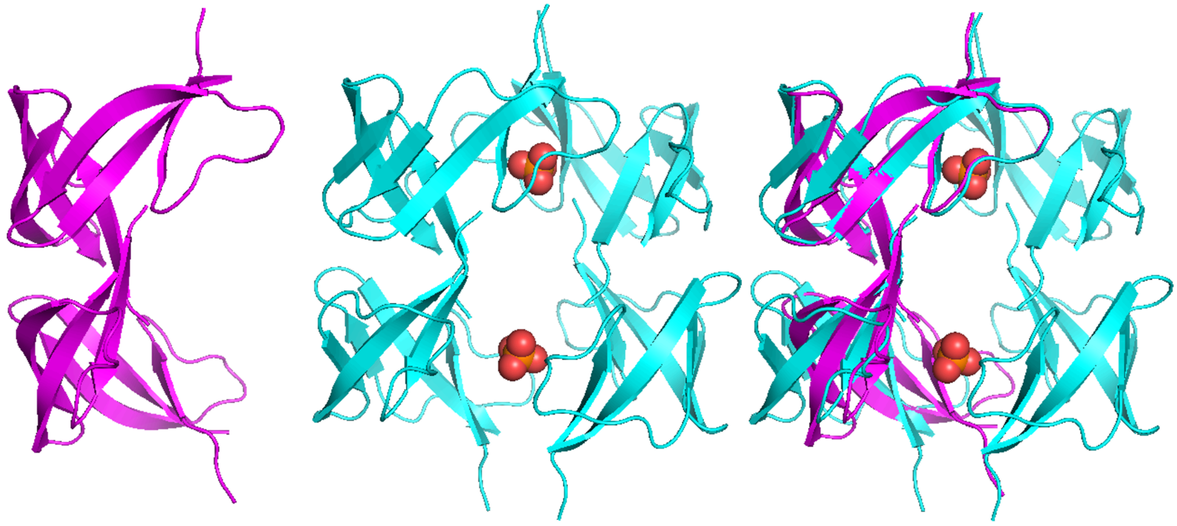

PDB: 1ZMQ, 4 chains　T1219v1o, 8 chains　Superimporsition

Figure S2. The comparison of 1ZMQ and current target (T1210v1o) structure. Both of them are the oligomers of human defensin-6. 1ZMQ is composed of 4 chains and solved by X-ray diffraction while the current target structure was solved by EM.

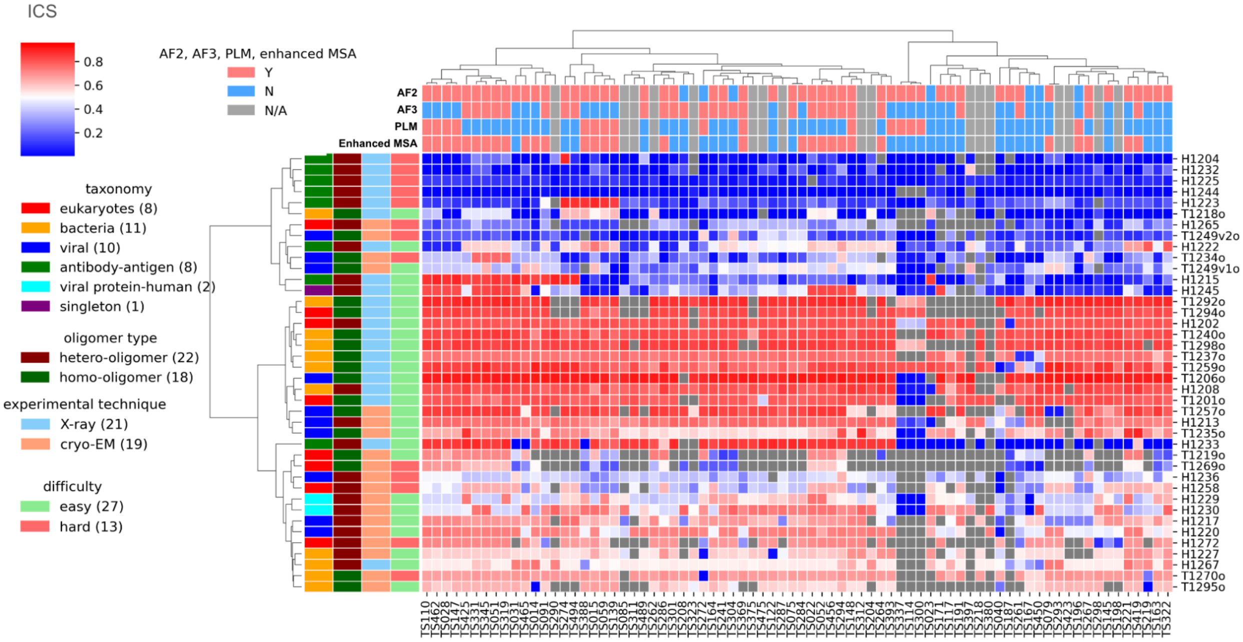

Figure S3. Overview of targets and participating groups with ICS. A) A heatmap of the performance of participating groups over Phase 1 oligomer targets. The value in each cell is the highest ICS value of one group for one target (Grey indicates that such a score is not available). The annotations on the x-axis and y-axis represent the different features of groups and targets, respectively. Only groups that submitted more than 25% of the models are included.

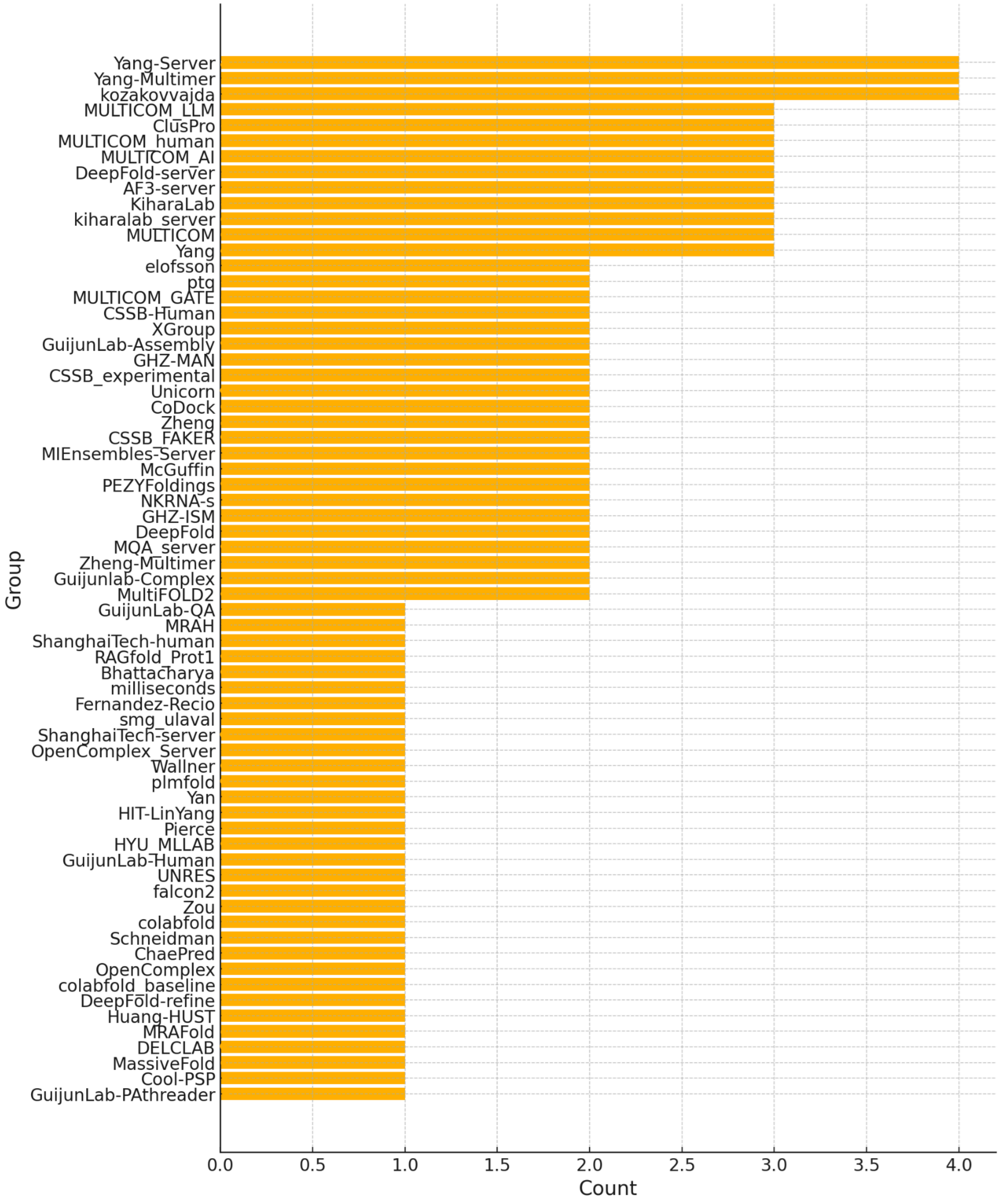

Figure S4 The count of difficult targets for which each group was able to predict models with DockQ ≥ 0.5. Groups were excluded if they failed to produce any models with DockQ ≥ 0.5 on difficult targets. The 13 difficult targets were defined based on Figure 2A and include those shown in Figures 2B–I as well as antibody/nanobody–antigen assemblies.

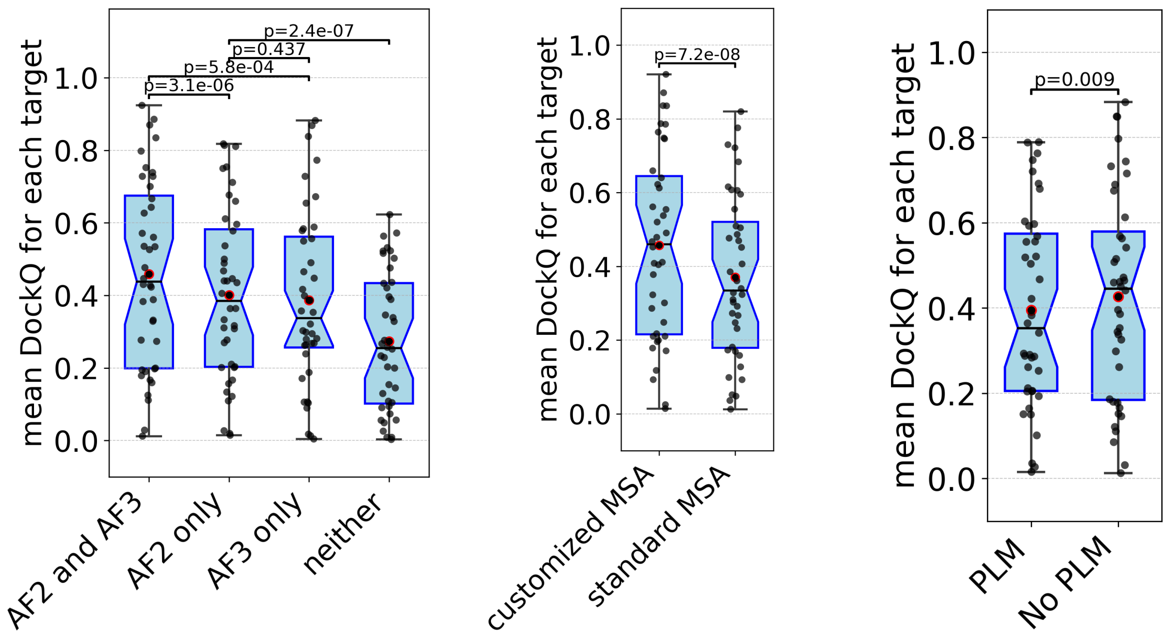

Figure S5. Relationship between methodologic features and prediction performance. Left: Groups categorized by use of AlphaFold2 (AF2) and/or AlphaFold3 (AF3). Middle: Groups using customized MSAs versus standard MSAs; Right: Groups using protein language models (PLMs) versus those that did not. For each target, the mean DockQ score of submitted models is shown. Groups incorporating both AF2 and AF3, customized MSAs, or PLMs tend to show higher performance.

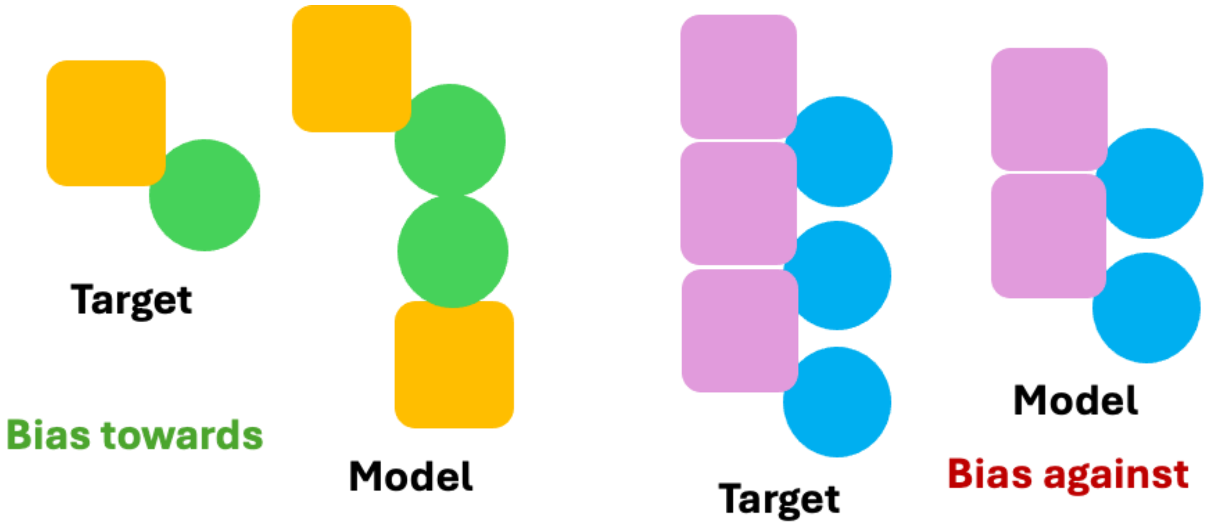

Figure S6. Scenarios where the OST pipeline will have undesirable bias. Left: a model with two incorrectly predicted interfaces, which will not be punished; right: a model with all the necessary interfaces to assemble into the target structure and no incorrectly predicted interfaces, which will be punished by OST due to missing chains appearing in the target.

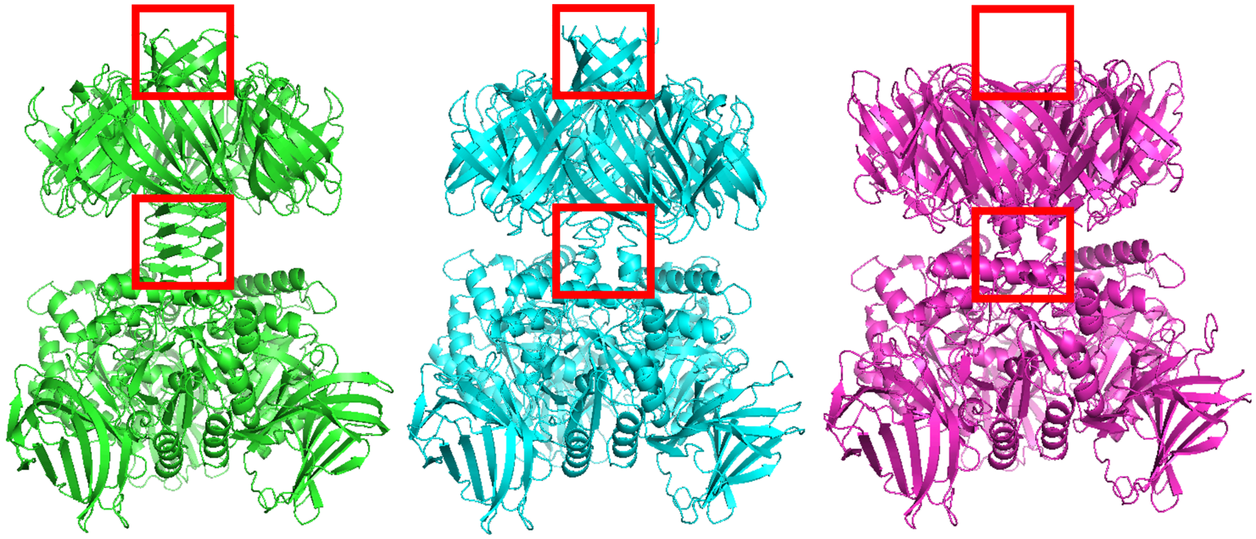

Figure S7. Structure of target H1236 and top-performing models. H1236 is a complex composed of six copies of gp30 and three copies of prokaryotic prokaryotic polysaccharide deacetylaseShown are the experimental structure of H1236 (left), the best model from KiharaLab (middle, DockQ = 0.615), and the best model from MassiveFold (right, DockQ = 0.379), the second-best performing group on this target. The red rectangular indicated regions participants usually failed to predict correct secondary structures and interactions.

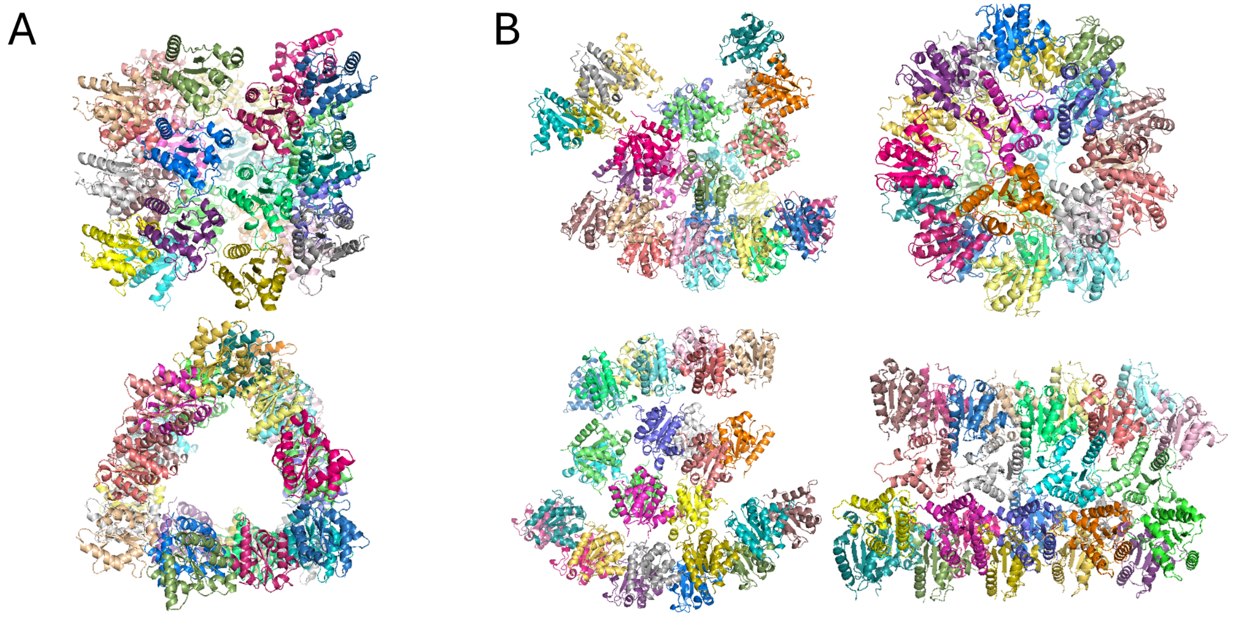

Figure S8. Structure of target H1265 and representative submitted models. (A) Experimental structure of H1265, a complex composed of TIR domains from TLR4 and MAL, shown from the side (top) and top-down (bottom) views. (B) Selected submitted models with either the relatively high TM-score, DockQ, or distinct alternative shape.

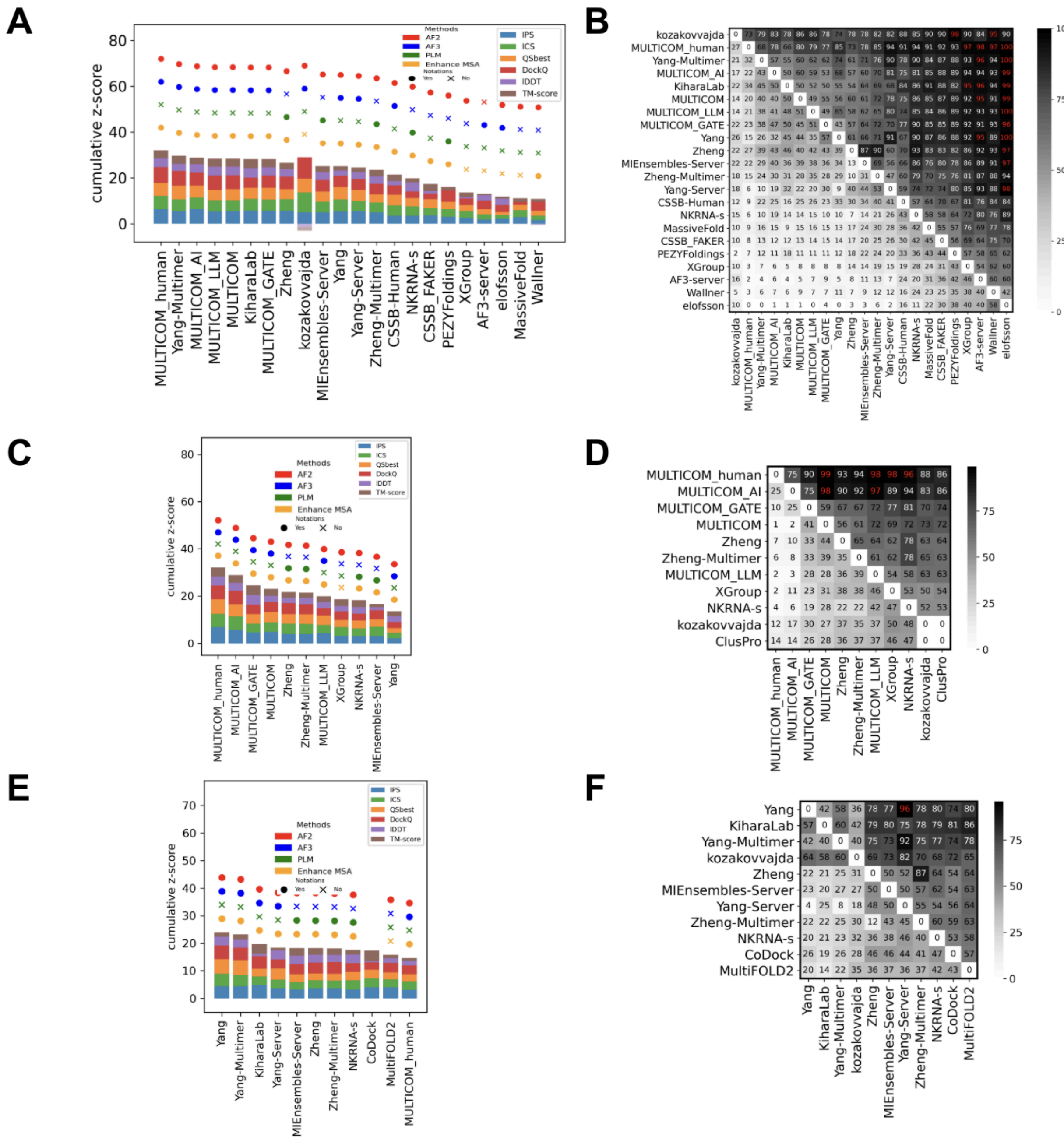

Figure S9. Rankings and head-to-head bootstrap comparison of top groups based on the first model for each group in phase 1 (A and B), phase 0 (C and D) and phase 2 (E and F).

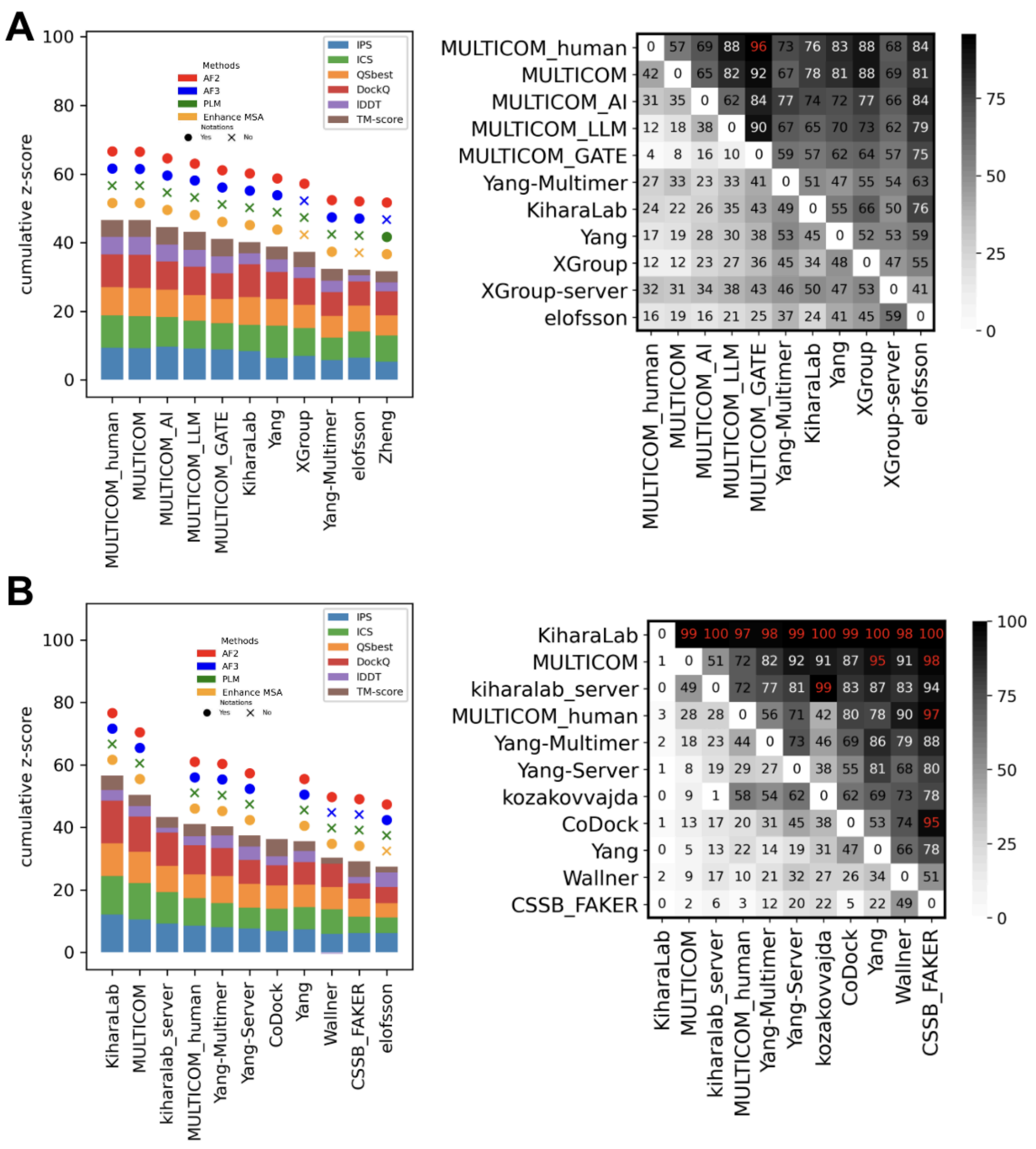

Figure S10. Rankings (left) and head-to-head bootstrap comparison (right) of top 11 groups based on the best model per group in phase 0 (A) and phase 2 (B).

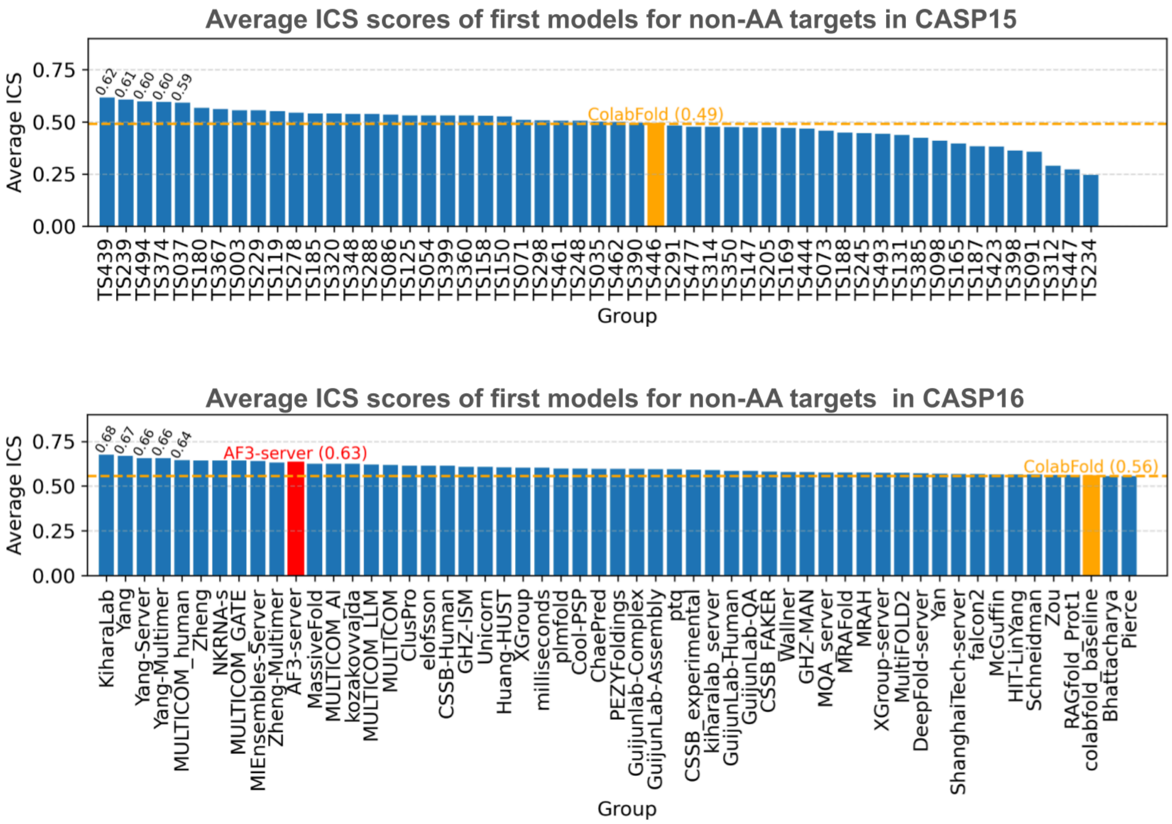

Figure S11. Average ICS for first models among top groups over normal targets (excluding AA and fiber targets) in CASP15 (top) and CASP16 (bottom), respectively.

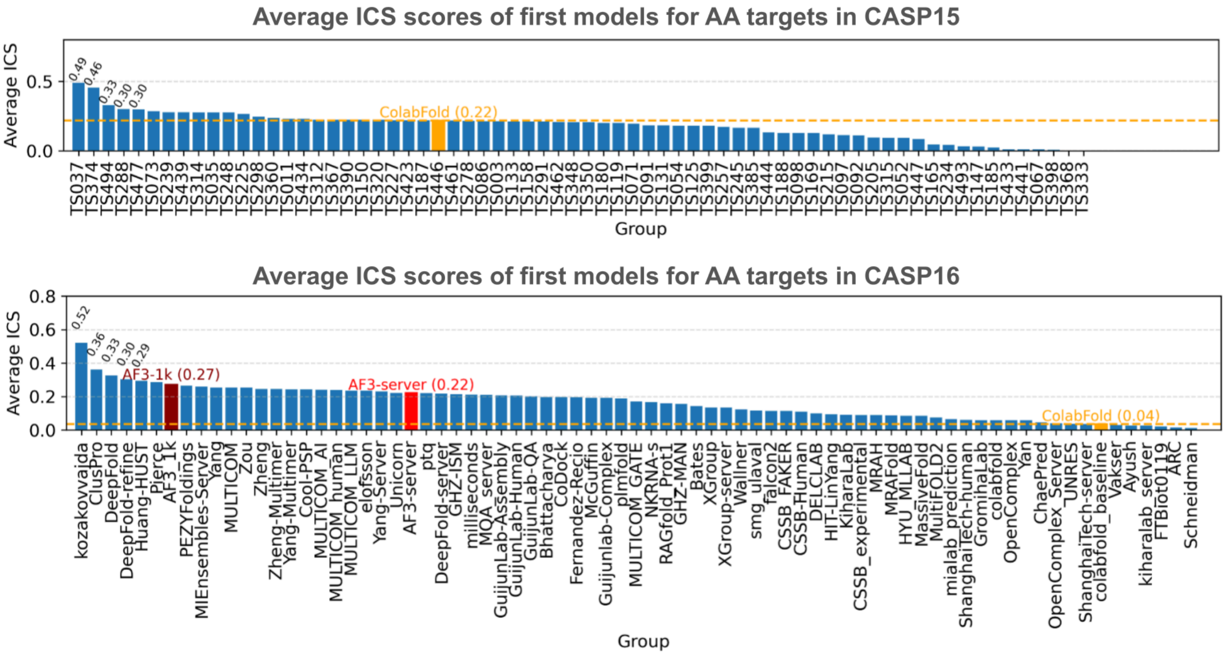

Figure S12. Average ICS for first models among top groups over AA targets in CASP15 (top) and CASP16 (bottom), respectively.

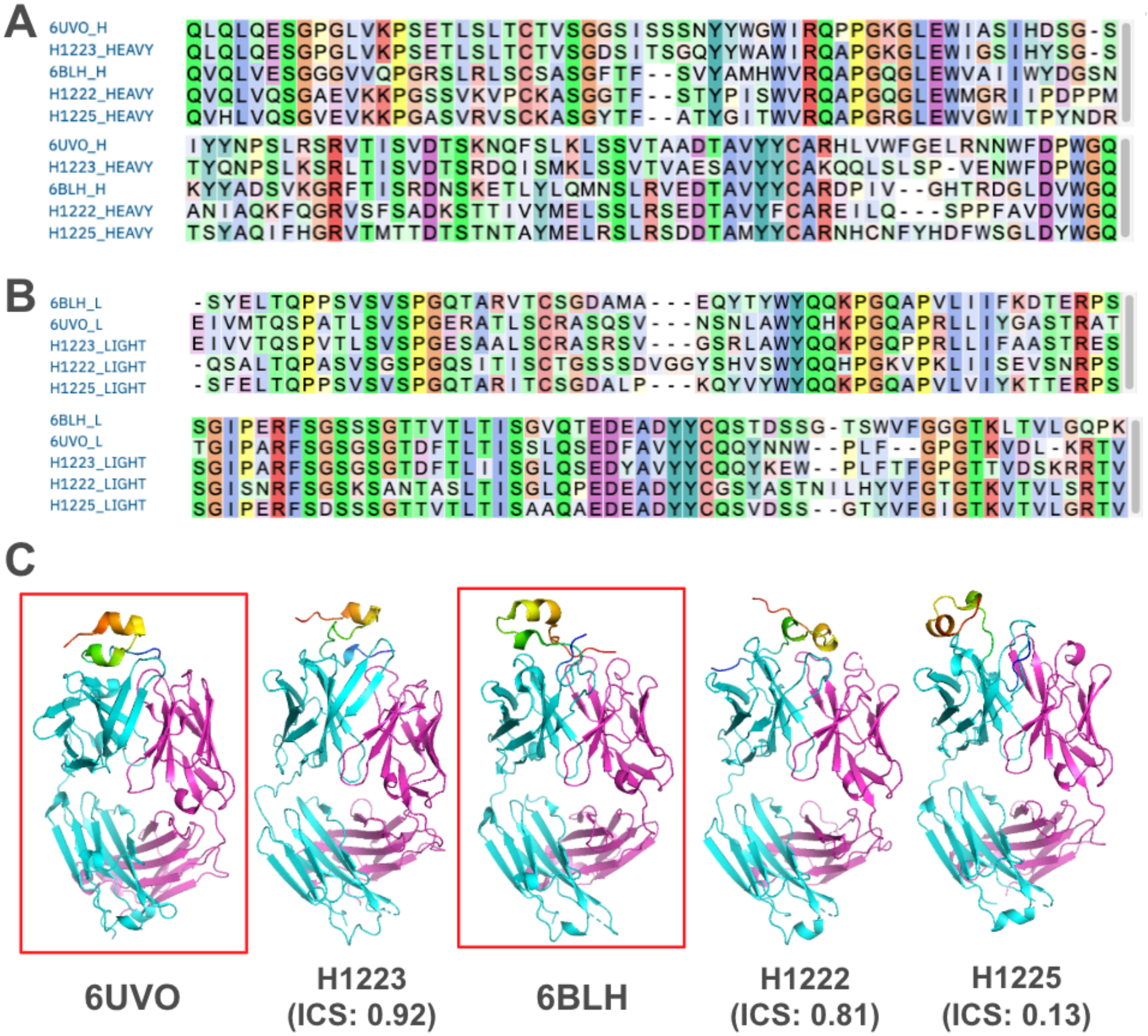

Figure S13. Homologous templates for antibody-antigen targets could not improve model quality due to high variability in antibody-antigen interfaces. Three oligomer targets, H1222, H1223, and H1225, all contain identical antigen sequences as two PDB templates 6UVO and 6BLH, which are complexes of this antigen with human antibodies. However, due to the high sequence variability in the complementarity-determining regions (CDRs) used for antigen binding in the antibodies, the interfaces between these antibodies and the antigens show large variability. Therefore, these homologous templates are not helpful in modeling these AA targets. **(A)** Sequence alignments containing the CDRs of heavy-chain antibodies in these targets and templates. **(B)** Sequence alignments containing the CDRs of light-chain antibodies in these targets and templates. **(C)** Different structures of the same antigen (on top and colored in rainbow from N- to C-terminus) bound to different human antibodies.

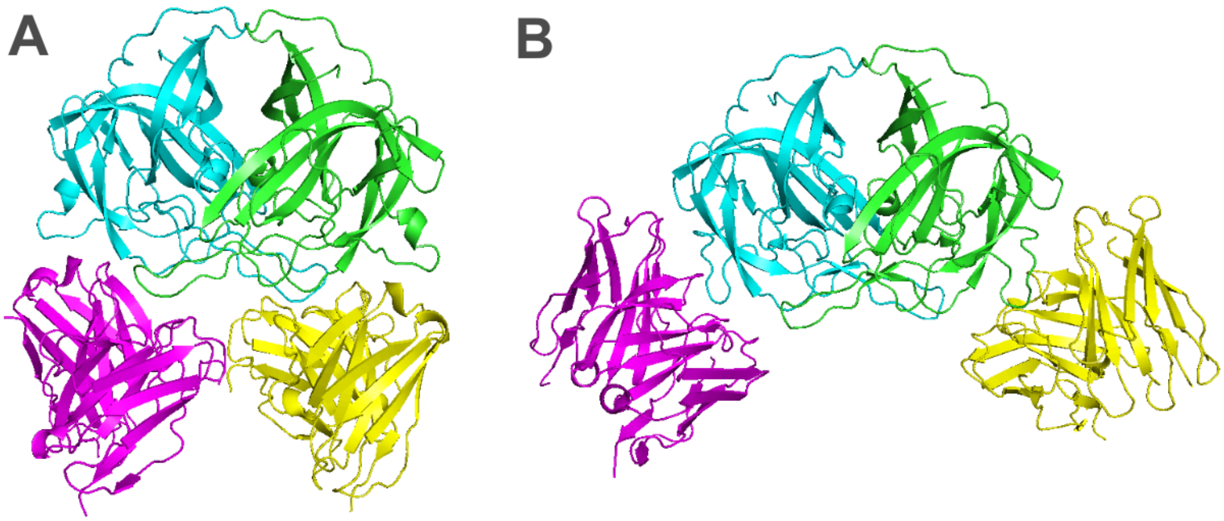

Figure S14. Homologous templates for antibody-antigen targets could not improve model quality due to high variability in antibody-antigen interfaces. (A) Target H1232; **(B)** its homologous template (5kov). Relying on this template will result in poor interface accuracy.
